# 5-(4-Chloro­phen­yl)-1*H*-tetra­zole

**DOI:** 10.1107/S1600536810003788

**Published:** 2010-03-03

**Authors:** Liang Xu, Li-Yan Gu, Dan-Yu Zhao, Bing Wang, Ting-Guo Kang

**Affiliations:** a College of Pharmacy, Liaoning University of Traditional Chinese Medicine, Dalian 116600, People’s Republic of China

## Abstract

The two independent mol­ecules of the title compound, C_7_H_5_ClN_4_, both lie on a twofold rotation axis that passes through the centroids of the five- and six-membered rings and the attached Cl C atom. One molecule is nearly planar [dihedral angle between rings = 0.22 (6)°], whereas the other is significantly twisted [dihedral angle = 17.38 (6)°]. In the crystal, adjacent mol­ecules are linked by N—H⋯N hydrogen bonds into a chain structure.

## Related literature

For the synthesis, see: Xu *et al.* (2009 ▶). For a related structure, see: Luo *et al.* (2006 ▶).
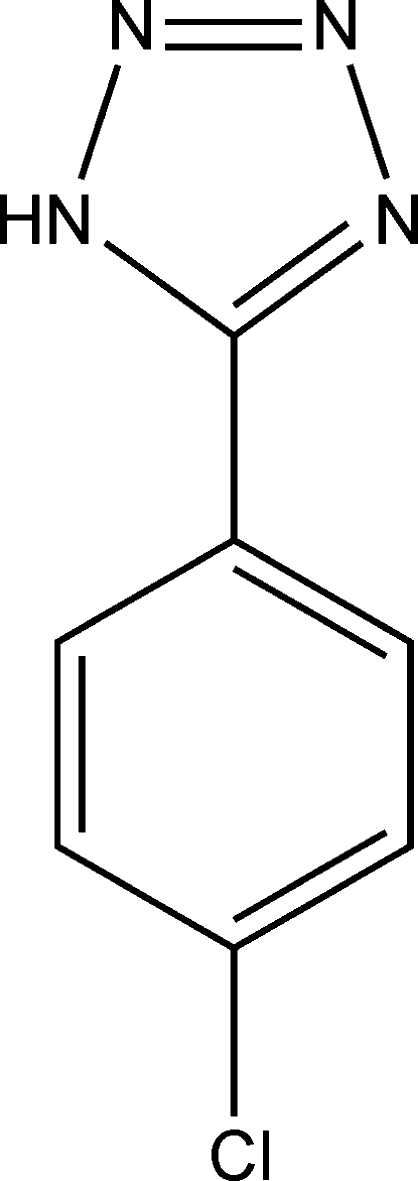


## Experimental

### 

#### Crystal data

C_7_H_5_ClN_4_
*M* *_r_* = 180.60
Monoclinic, 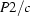

*a* = 9.4596 (19) Å
*b* = 11.437 (2) Å
*c* = 7.2988 (15) Å
β = 107.91 (3)°
*V* = 751.4 (3) Å^3^
*Z* = 4
Mo *K*α radiation
μ = 0.45 mm^−1^
*T* = 291 K
0.21 × 0.14 × 0.11 mm

#### Data collection

Rigaku R-AXIS RAPID diffractometer
Absorption correction: multi-scan (*ABSCOR*; Higashi, 1995 ▶) *T*_min_ = 0.912, *T*_max_ = 0.952
7237 measured reflections
1720 independent reflections
1194 reflections with *I* > 2σ(*I*)
*R*_int_ = 0.038

#### Refinement

*R*[*F* ^2^ > 2σ(*F* ^2^)] = 0.050
*wR*(*F* ^2^) = 0.135
*S* = 1.05
1720 reflections
118 parameters
H atoms treated by a mixture of independent and constrained refinement
Δρ_max_ = 0.77 e Å^−3^
Δρ_min_ = −0.24 e Å^−3^

### 

Data collection: *RAPID-AUTO* (Rigaku, 1998 ▶); cell refinement: *RAPID-AUTO*; data reduction: *CrystalClear* (Rigaku/MSC, 2002 ▶); program(s) used to solve structure: *SHELXS97* (Sheldrick, 2008 ▶); program(s) used to refine structure: *SHELXL97* (Sheldrick, 2008 ▶); molecular graphics: *SHELXTL* (Sheldrick, 2008 ▶); software used to prepare material for publication: *SHELXL97*.

## Supplementary Material

Crystal structure: contains datablocks I, global. DOI: 10.1107/S1600536810003788/ng2726sup1.cif

Structure factors: contains datablocks I. DOI: 10.1107/S1600536810003788/ng2726Isup2.hkl

Additional supplementary materials:  crystallographic information; 3D view; checkCIF report

## Figures and Tables

**Table 1:** Hydrogen-bond geometry (Å, °)

*D*—H⋯*A*	*D*—H	H⋯*A*	*D*⋯*A*	*D*—H⋯*A*
N1—H3⋯N3	0.85 (1)	2.05 (1)	2.889 (2)	172 (1)
N3—H6⋯N1	0.83 (3)	2.08 (4)	2.889 (2)	165.7 (2)

**Fig. 1.**
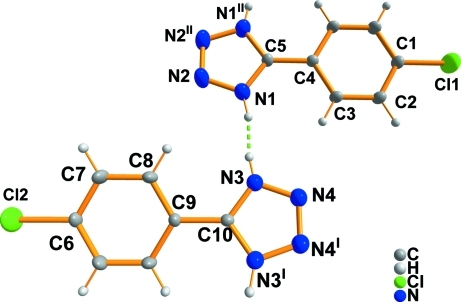
The molecular structure of the title compound, showing displacement ellipsoids at the 50% probability level for non-H atoms. Dashed lines indicate the hydrogen bonds.

**Fig. 2.**
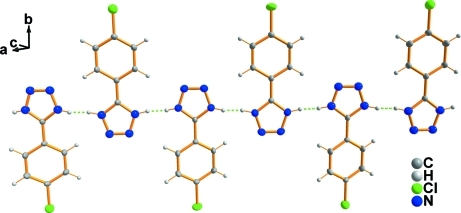
A partial packing view, showing one-dimensional chain structure. Dashed lines indicate the hydrogen bonds.

## supplementary crystallographic information

### Comment

The tetrazole functional group attracted considerable attention over recent years, because of both the intriguing architectures in coordination chemistry and the potential applications in medicinal chemistry and materials science (Luo *et al.*, 2006). Herein, we reported the synthesis and the crystal structure of the title compound.

In the asymmetric unit of the title compound, C_7_H_5_ClN_4_, contains two half molecules of 5-(4-Chlorophenyl)-1*H*-tetrazole. In these two molecules, the centres of bezene and tetrazole rings locate on the symmetry plane, with the dihedral angle of 0.22 (6)° and 17.38 (6)°, respectively.

A one-dimensional chain structure is built up by N—H···N hydrogen bonds between the imino groups of the title compound.

### Experimental

For the preparation of the title compound, 4-chlorobenzonitrile (13.7 g, 0.10 mol), ammonium chloride (13.4 g, 0.25 mmol) and NaN3 (7.8 g, 0.12 mol) were dissolved in DMF (120 ml). The mixture was heated to reflux stirred for 24 h under stirring. Then, it was cooled to room temperature and poured into cold water and acidified to pH = 2 with concentrated hydrochloric acid. The suspension was filtrated, and the residue was washed with water and ethanol for several times, and then dried (11.1 g, 61.8 %). Crystals suitable for X-ray analysis were obtained by recrystallization in the EtOH solution.

### Refinement

Due to the title compound molecules located on the symmetry planes, the H atoms bound to N atoms were disordered into two positions with the occupancies of 0.5, respectively. H atoms bound to N atoms were located in a difference Fourier map and refined freely. H atoms bound to C atoms were placed in calculated positions and treated as riding on their parent atoms, with C—H = 0.93 Å (aromatic) and U_iso_(H) = 1.2U_eq_(C).

### Crystal data

**Table:**

C_7_H_5_ClN_4_	*F*(000) = 368
*M**_r_* = 180.60	*D*_x_ = 1.596 Mg m^−^^3^
Monoclinic, *P*2/*c*	Mo *K*α radiation, λ = 0.71073 Å
Hall symbol: -P 2yc	Cell parameters from 5352 reflections
*a* = 9.4596 (19) Å	θ = 3.1–27.5°
*b* = 11.437 (2) Å	µ = 0.45 mm^−^^1^
*c* = 7.2988 (15) Å	*T* = 291 K
β = 107.91 (3)°	Block, colorless
*V* = 751.4 (3) Å^3^	0.21 × 0.14 × 0.11 mm
*Z* = 4	

### Data collection

**Table:**

Rigaku R-AXIS RAPID diffractometer	1720 independent reflections
Radiation source: fine-focus sealed tube	1194 reflections with *I* > 2σ(*I*)
graphite	*R*_int_ = 0.038
ω scan	θ_max_ = 27.5°, θ_min_ = 3.4°
Absorption correction: multi-scan (*ABSCOR*; Higashi, 1995)	*h* = −12→12
*T*_min_ = 0.912, *T*_max_ = 0.952	*k* = −14→14
7237 measured reflections	*l* = −9→9

### Refinement

**Table:**

Refinement on *F*^2^	Primary atom site location: structure-invariant direct methods
Least-squares matrix: full	Secondary atom site location: difference Fourier map
*R*[*F*^2^ > 2σ(*F*^2^)] = 0.050	Hydrogen site location: inferred from neighbouring sites
*wR*(*F*^2^) = 0.135	H atoms treated by a mixture of independent and constrained refinement
*S* = 1.05	*w* = 1/[σ^2^(*F*_o_^2^) + (0.0807*P*)^2^] where *P* = (*F*_o_^2^ + 2*F*_c_^2^)/3
1720 reflections	(Δ/σ)_max_ < 0.001
118 parameters	Δρ_max_ = 0.77 e Å^−^^3^
0 restraints	Δρ_min_ = −0.24 e Å^−^^3^

### Special details

**Table:**

Geometry. All esds (except the esd in the dihedral angle between two l.s. planes) are estimated using the full covariance matrix. The cell esds are taken into account individually in the estimation of esds in distances, angles and torsion angles; correlations between esds in cell parameters are only used when they are defined by crystal symmetry. An approximate (isotropic) treatment of cell esds is used for estimating esds involving l.s. planes.
Refinement. Refinement of *F*^2^ against ALL reflections. The weighted *R*-factor wR and goodness of fit *S* are based on *F*^2^, conventional *R*-factors *R* are based on *F*, with *F* set to zero for negative *F*^2^. The threshold expression of *F*^2^ > σ(*F*^2^) is used only for calculating *R*-factors(gt) etc. and is not relevant to the choice of reflections for refinement. *R*-factors based on *F*^2^ are statistically about twice as large as those based on *F*, and *R*- factors based on ALL data will be even larger.

### Fractional atomic coordinates and isotropic or equivalent isotropic displacement parameters (Å^2^)

**Table:**

	*x*	*y*	*z*	*U*_iso_*/*U*_eq_	Occ. (<1)
C1	0.0000	0.7724 (3)	0.2500	0.0340 (7)	
C2	0.1238 (2)	0.7123 (2)	0.3612 (3)	0.0375 (5)	
H1	0.2067	0.7530	0.4358	0.045*	
C3	0.1235 (2)	0.59197 (19)	0.3606 (3)	0.0331 (5)	
H2	0.2068	0.5518	0.4350	0.040*	
C4	0.0000	0.5293 (3)	0.2500	0.0282 (6)	
C5	0.0000	0.4029 (3)	0.2500	0.0288 (6)	
C6	0.5000	−0.0727 (3)	0.7500	0.0296 (6)	
C7	0.3680 (2)	−0.0135 (2)	0.6704 (3)	0.0389 (5)	
H4	0.2797	−0.0544	0.6188	0.047*	
C8	0.3688 (2)	0.1065 (2)	0.6683 (3)	0.0354 (5)	
H5	0.2809	0.1471	0.6119	0.042*	
C9	0.5000	0.1681 (2)	0.7500	0.0258 (6)	
C10	0.5000	0.2975 (3)	0.7500	0.0267 (6)	
Cl1	0.0000	0.92391 (7)	0.2500	0.0492 (3)	
Cl2	0.5000	−0.22534 (7)	0.7500	0.0464 (3)	
N1	0.1109 (2)	0.33285 (16)	0.3488 (2)	0.0349 (4)	
H3	0.1934	0.3493	0.4318	0.050 (15)*	0.50
N2	0.0660 (2)	0.22079 (17)	0.3089 (3)	0.0418 (5)	
N3	0.3941 (2)	0.36603 (16)	0.6406 (3)	0.0349 (4)	
H6	0.322 (5)	0.352 (4)	0.545 (6)	0.016 (9)*	0.50
N4	0.4364 (2)	0.47685 (17)	0.6842 (3)	0.0398 (5)	

### Atomic displacement parameters (Å^2^)

**Table:**

	*U*^11^	*U*^22^	*U*^33^	*U*^12^	*U*^13^	*U*^23^
C1	0.0332 (14)	0.0274 (17)	0.0377 (15)	0.000	0.0056 (12)	0.000
C2	0.0313 (10)	0.0320 (12)	0.0424 (11)	−0.0035 (8)	0.0011 (9)	−0.0024 (9)
C3	0.0270 (9)	0.0298 (12)	0.0349 (10)	0.0000 (8)	−0.0016 (8)	0.0017 (8)
C4	0.0267 (13)	0.0285 (17)	0.0270 (13)	0.000	0.0046 (11)	0.000
C5	0.0305 (13)	0.0261 (15)	0.0266 (12)	0.000	0.0038 (11)	0.000
C6	0.0365 (14)	0.0215 (15)	0.0272 (13)	0.000	0.0047 (12)	0.000
C7	0.0324 (11)	0.0310 (12)	0.0456 (12)	−0.0054 (8)	0.0006 (10)	−0.0066 (9)
C8	0.0242 (9)	0.0328 (12)	0.0408 (11)	0.0009 (8)	−0.0021 (8)	−0.0002 (9)
C9	0.0292 (13)	0.0219 (15)	0.0238 (12)	0.000	0.0044 (11)	0.000
C10	0.0248 (12)	0.0282 (16)	0.0244 (12)	0.000	0.0037 (11)	0.000
Cl1	0.0465 (5)	0.0241 (5)	0.0709 (6)	0.000	0.0090 (4)	0.000
Cl2	0.0591 (5)	0.0225 (5)	0.0503 (5)	0.000	0.0061 (4)	0.000
N1	0.0332 (8)	0.0275 (10)	0.0356 (9)	0.0027 (7)	−0.0019 (8)	0.0001 (7)
N2	0.0437 (10)	0.0245 (10)	0.0456 (10)	0.0031 (8)	−0.0036 (8)	0.0027 (8)
N3	0.0329 (9)	0.0257 (10)	0.0374 (9)	0.0008 (7)	−0.0018 (8)	0.0000 (7)
N4	0.0381 (9)	0.0259 (10)	0.0450 (10)	0.0016 (8)	−0.0027 (8)	0.0002 (8)

### Geometric parameters (Å, °)

**Table:**

C1—C2	1.385 (3)	C7—C8	1.373 (3)
C1—C2^i^	1.385 (3)	C7—H4	0.9300
C1—Cl1	1.732 (3)	C8—C9	1.392 (2)
C2—C3	1.376 (3)	C8—H5	0.9300
C2—H1	0.9300	C9—C8^ii^	1.392 (2)
C3—C4	1.397 (3)	C9—C10	1.480 (4)
C3—H2	0.9300	C10—N3^ii^	1.328 (3)
C4—C3^i^	1.397 (3)	C10—N3	1.328 (3)
C4—C5	1.446 (4)	N1—N2	1.353 (3)
C5—N1^i^	1.340 (3)	N1—H3	0.8492
C5—N1	1.340 (3)	N2—N2^i^	1.280 (4)
C6—C7^ii^	1.382 (3)	N3—N4	1.338 (3)
C6—C7	1.382 (3)	N3—H6	0.83 (4)
C6—Cl2	1.746 (3)	N4—N4^ii^	1.288 (3)
			
C2—C1—C2^i^	120.5 (3)	C8—C7—H4	120.4
C2—C1—Cl1	119.77 (15)	C6—C7—H4	120.4
C2^i^—C1—Cl1	119.77 (15)	C7—C8—C9	120.55 (19)
C3—C2—C1	119.6 (2)	C7—C8—H5	119.7
C3—C2—H1	120.2	C9—C8—H5	119.7
C1—C2—H1	120.2	C8—C9—C8^ii^	119.2 (3)
C2—C3—C4	121.05 (19)	C8—C9—C10	120.38 (13)
C2—C3—H2	119.5	C8^ii^—C9—C10	120.38 (14)
C4—C3—H2	119.5	N3^ii^—C10—N3	107.6 (3)
C3—C4—C3^i^	118.2 (3)	N3^ii^—C10—C9	126.18 (13)
C3—C4—C5	120.88 (14)	N3—C10—C9	126.18 (13)
C3^i^—C4—C5	120.88 (14)	C5—N1—N2	107.97 (18)
N1^i^—C5—N1	106.6 (3)	C5—N1—H3	130.3
N1^i^—C5—C4	126.70 (14)	N2—N1—H3	121.5
N1—C5—C4	126.70 (14)	N2^i^—N2—N1	108.73 (11)
C7^ii^—C6—C7	121.3 (3)	C10—N3—N4	107.51 (18)
C7^ii^—C6—Cl2	119.34 (14)	C10—N3—H6	131 (3)
C7—C6—Cl2	119.34 (14)	N4—N3—H6	120 (3)
C8—C7—C6	119.1 (2)	N4^ii^—N4—N3	108.67 (11)

Symmetry codes: (i) −*x*, *y*, −*z*+1/2; (ii) −*x*+1, *y*, −*z*+3/2.

### Hydrogen-bond geometry (Å, °)

**Table:**

*D*—H···*A*	*D*—H	H···*A*	*D*···*A*	*D*—H···*A*
N1—H3···N3	0.85 (1)	2.05 (1)	2.889 (2)	172 (1)
N3—H6···N1	0.83 (3)	2.08 (4)	2.889 (2)	165.7 (2)
